# The Ameliorative Effect of Thymoquinone on Vincristine-Induced Peripheral Neuropathy in Mice by Modulating Cellular Oxidative Stress and Cytokine

**DOI:** 10.3390/life13010101

**Published:** 2022-12-29

**Authors:** Sattam Khulaif Alenezi

**Affiliations:** Department of Pharmacology and Toxicology, Unaizah College of Pharmacy, Qassim University, Qassim 51452, Saudi Arabia; sk.alenezi@qu.edu.sa

**Keywords:** thymoquinone, vincristine, peripheral neuropathy, mice, cellular oxidative stress, cytokine

## Abstract

Thymoquinone (TQ), an active constituent of *Nigella sativa*, has been reported to exert a broad spectrum of pharmacological effects, including neuroprotective, anticancer, anti-inflammatory, antidiabetic, antiepileptic, antioxidant, and other modulatory roles in inflammation in experimental studies. The present study aims to evaluate the potential effects of TQ on vincristine-induced neuropathy in mice, as well as the possible role of oxidative stress, and pro- and anti-inflammatory cytokine in neuropathy development. A Swiss strain of male albino mice were randomly divided into seven groups, comprising of five animals each. Vincristine sulfate (0.1 mg/kg, i.p.) was administered for 10 consecutive days for the induction of peripheral neuropathy. The animals received their respective treatment of TQ (2.5, 5, and 10 mg/kg, p.o.) and pregabalin (10 mg/kg, p.o.) concurrently with vincristine for 10 days followed by 4 days post treatment. The animals were assessed for pain and related behavior on day 7 and 14 using hot and cold plates, and a rotarod test. TQ preventive treatment attenuated vincristine induced neuropathy in a dose dependent manner evidenced as a significant (*p* < 0.001) increase in reaction time on the hot plate and the cold plate, and a fall off time on the rotarod test. Further, TQ preventive treatment resulted in a significant (*p* < 0.001) reduction in the number of flinches and duration of paw elevation in a formalin test. Preventative treatment with TQ abolished the vincristine-induced rise in malondialdehyde and glutathione depletion in sciatic nerve tissue, as well as the blood IL-6 levels. In conclusion, TQ at 2.5, 5, and 10 mg/kg dose produced significant attenuation of neuropathic pain induced by vincristine which may be due to its antinociceptive, antioxidant, and anti-proinflammatory activity.

## 1. Introduction

Peripheral neuropathy is a frequently experienced disabling condition, often limiting the therapeutic effectiveness of vinca alkaloids, platinum-based compounds, taxanes, and other anticancer drugs. Besides the psychosocial issues, such as anxiety, depression, distress, and disruption of family life and/or routine work following a cancer diagnosis, the therapeutic intervention with various chemotherapeutic agents often produces debilitating adverse effects in cancer patients. According to an estimate, approximately 30–40% of patients experience neuropathy following anticancer treatment [1]. The reported occurrence of neuropathy due to chemotherapy is as high as 68%, 60%, and 30% at one, three, and six months, respectively, following the administration of chemotherapy [2]. Although chemotherapy-induced peripheral neuropathy (CIPN) is generally considered a reversible condition as the neurotoxic effects are mainly produced on the peripheral nervous system, there is, however, increasing evidence suggesting its lifelong persistence in cancer survivors.

Vincristine (VCR), an alkaloid, is one of the widely used anticancer drugs, primarily indicated for the management of tumors such as acute lymphocytic leukemia, chronic myeloid leukemia, sarcoma, and primary brain tumors [3], the therapy of which often produces dose and duration dependent peripheral neuropathy with its administration [4], thus needing dose reduction or sometimes the discontinuation of therapy. Clinically, it is manifested as the dysfunction in sensory, motor, and autonomic neurons. The symptoms of sensory dysfunctions are most frequent and typically develop within several weeks of therapy with vincristine, manifesting as hyperalgesia, paresthesia, dysesthesia, allodynia, and spontaneous pain [5]. VCR-induced impairment of motor neuron function includes weakness, incoordination, and disturbed gait; in addition, it also produces alterations in autonomic functions evidenced by altered thermoregulation, blood pressure, and intestinal motility [3]. Despite this, the exact pathophysiologic mechanism of neuropathy due to chemotherapeutic agents is not clearly understood. However, several putative pathologic mechanisms have been proposed, including sensory terminal degeneration in the skin due to impairment of microtubule function, alterations in intracellular signaling pathway, neurohumoral transmission, excitability, receptors, and metabolism of the cells by mitochondrial dysfunction of neurons [6].

A growing body of literature reported the contribution of mitochondrial reactive oxygen species in the pathogenesis pain-related behavior in experimental animals [7]. The relatively low antioxidant capacity and high oxygen demand of nerve cells counterfeit the high vulnerability of neurons to oxidative attack. It has been reported that VCR-induced toxicity is at least partly mediated by oxidative stress, induction of inflammation in cells, and activation of the mitochondrial apoptotic pathway in the kidney [8]. Interleukin-6 (IL-6) is a biomolecule with pro- and anti-inflammatory properties that plays a variety of pathophysiological roles. It exerts many positive effects, such as increased blood flow and regulation of metabolism, suppression of long-term inflammatory processes, and regeneration of peripheral neurons, contrary to its pro-inflammatory effects. The anti-inflammatory effects of IL-6 are mainly through classic signaling by activation of cell membrane bound IL-6 receptors restricted to certain cells only, whereas the pro-inflammatory effects of IL-6 are mediated through trans-signaling by soluble IL-6 receptors using gp130 protein [9]. Interestingly, there was a significant reduction in diabetes-induced systemic oxidative stress following the inhibition of pro-inflammatory trans-signaling of IL-6 [10]. Therefore, it is plausible that the understanding of the possible role of systemic IL-6 in the neuropathogenic mechanism can decipher the implication of IL-6 classical signaling in the immune response and repair process of inflammatory diseases.

Despite the availability of a wide range of analgesics, both peripherally and centrally acting drugs were unsuccessful in reverting the vincristine-induced peripheral neuropathy, leading to further deterioration of cancer patients’ life and general wellbeing [11]. However, the wide spectrum of side effects with tricyclic antidepressants and anticonvulsant limits their usefulness in neuropathic pain due to vincristine therapy [12]. Accordingly, intense efforts have been put forth, directed toward the search for novel therapeutic agents and further elucidation of pathophysiologic mechanisms involved in vincristine-induced peripheral neuropathy. Since antiquity, herbs and drugs of herbal origin have been in use for managing various disorders, including pains of neuronal origin [13] and have evolved as a distinct area of research for novel therapeutic agents from natural sources. There are some clinical reports that have advocated for the encouraging effects of herbal drugs on neuropathic pain [14,15].

*Nigella sativa* Linn. (Ranunculaceae) is one of several herbal drugs that have shown promising results in animal models of experimentally induced neuropathic pain [16], as well as antioxidants against doxorubicin-induced oxidative stress in mice [17]. Thymoquinone (TQ) from *Nigella sativa* is a bioactive compound that has been extensively studied for its many pharmacological actions, such as antioxidant, anti-inflammatory, cytoprotective, immunomodulatory, antineoplastic, antibacterial, antidiabetic, and analgesic effects [18,19,20]. In addition, TQ offers various beneficial effects which are pertinent to neurological disorders, including antiepileptic [21], antianxiety [22], antidepressant [23], antipsychotic, and the amelioration of memory impairment [24], and thus improves the quality of life in neuropathic patients. TQ treatment has shown an ameliorative effect on pain originating from neurodegeneration induced by mechanical injury to the spinal cord in rats [25]. Furthermore, TQ alleviated the STZ-induced morphological changes and myelin breakdown in the sciatic nerves of diabetic rats [26]. Previous studies have underscored reduction of vincristine induced neuropathy scores with TQ, however, there remains dearth in research finding conclusive evidence on its ameliorative efficacy in the vincristine-induced neuropathy and also its possible role in oxidative stress and cytokine.

Therefore, it would be quite interesting to explore the possible effects of TQ in neuropathic pain induced by vincristine in mice. In addition, the study investigated the possible role of oxidative stress and cytokines with dual pro- and anti-inflammatory functions in neuropathy, as well as the modulatory effects of TQ in mice with VCR-induced neuropathy. The study’s findings will undoubtedly be useful for lead optimization and will provide scientific evidence for the potential medicinal use of *Nigella sativa* and its active ingredient in treating human ailments.

## 2. Materials and Methods

### 2.1. Animals

A Swiss strain of male albino mice (25–30 g) were procured from the animal house facility of Qassim University, Saudi Arabia. The mice were housed in polypropylene cages with 5 animals each under a standard circadian cycle, and maintained at 25 ± 2 °C. The animals were allowed free access of pellet feed supplied from a local animal feed vendor for rat and mice, and water was provided ad libitum.

### 2.2. Drugs and Chemicals

Vincristine was obtained from the Oncology center of King Fahad Specialist Hospital, Buraidah, Saudi Arabia. Thymoquinone was purchased from Sigma Aldrich, St. Louis, MO, USA. Serum IL-6 levels were estimated by using a commercially available ELISA kit. All the drugs and chemicals used in the current study for biochemical estimation of oxidative stress parameters were of analytical grade.

### 2.3. Experimental Protocol

Thirty-five Swiss strain male albino mice of 8–12 weeks, weighing between 25–30 g, were segregated into seven groups of 5 animals each. The Vincristine sulfate was injected intraperitoneally (i.p.), while thymoquinone (TQ) was administered orally by a gavage needle. Vincristine was diluted with sterile water for injection. TQ was dissolved in distilled water using 2.5% dimethyl sulfoxide (DMSO), whereas pregabalin was suspended in 0.5% carboxymethylcellulose in distilled water. Group I (normal control) animals were treated with vehicle (2.5% dimethyl sulfoxide (DMSO) in distilled water; 10 mL/kg, p.o.) once daily for 14 days. Group II (pathogenic control) received VCR (0.1 mg/kg, i.p.) once daily for 10 days [16], followed by vehicle for 4 days. The animals of group III–V received concurrent treatment of different TQ doses ranging from 2.5, 5, and 10 mg/kg orally through an oral gavage needle and VCR (0.1 mg/kg, i.p.) once daily for 10 consecutive days, followed by TQ post-treatment for 4 days [27]. The animals of group VI (positive control) were concurrently treated with VCR (0.1 mg/kg, i.p.) and pregabalin (PGB, 10 mg/kg, p.o.) once daily for 10 days, followed by post-treatment with PGB for 4 days. Groups VII (per se) were treated with TQ (10 mg/kg, p.o.) alone once daily for 14 days. The animals were assessed for neuropathic pain and related behavior on a hot plate and cold plate, and by a rotarod test on day 7 of drug treatment. After completion of treatment schedule on day 14, the animals were again assessed for neuropathic pain and then subjected to a formalin test. The animals were anesthetized with diethyl ether and blood samples were collected for the separation of serum and estimations of serum IL-6 levels. The mice were then sacrificed for the isolation of sciatic nerves, and the estimation of lipid peroxidation and oxidative parameters were carried out on the sciatic nerve tissue. 

### 2.4. Behavioral Assessment

#### 2.4.1. Rotarod Test

Mice were placed individually on a rotating rod, with dimensions of 3 cm diameter and 30 cm long with a rough surface, mounted over 15 cm above the base rotating at a speed of 24 rpm, after 30 mins of respective drug treatment with a 300 s cutoff time. The interval between the mounting of the animals on the rotating bar and fall off time were recorded as the response as described in an earlier study by Kuribara et al. [28].

#### 2.4.2. Hot Plate Test

The test was conducted by a procedure previously reported by Tiwari et al. (2011). Briefly, each mouse was placed individually on a hot plate (Eddy’s hot plate) with a preset temperature of 55 ± 1 °C. The mice were observed for latency to paw licking or jump response as a first sign of pain reflex against thermally induced algesia. The animals were allowed on the hotplate for a maximum of 10 s to avoid damage to the paw [29].

#### 2.4.3. Cold Plate Test

In the cold plate test paradigm, each mouse was individually put on an ice-cold platform which was dipped 1 cm below in cold water maintained at 4 °C. The animals were placed in such a way that the mouse’s skin remained in cold water. The animals were observed for latency of paw licking, paw movement, and escape as a response to cold induced algesia. The animals were kept for a maximum period of 30 s on the ice-cold platform to avoid unnecessary harm to the animal [30].

#### 2.4.4. Formalin Test

The animals were subjected to formalin test at the end of the study. In brief, each mouse was allowed to explore the observation box for acclimatization before commencement of the test. Each animal was allowed 15 min for the acclimatization period. The animals were administered 50 mL of 2.5% formalin in the intraplantar region of the hind paw. The animals were observed for paw licking duration and number of flinches for quantification of nociception during a period of the first 10 min (acute phase) and then at 20–40 min (delayed phase) [31].

### 2.5. Biochemical Estimation

The serum IL-6 level was measured by a commercially available mouse ELISA kit. The estimation of malondialdehyde (MDA) and reduced glutathione (GSH) levels in 10% sciatic nerve tissue homogenate in tris buffer was measured following the methods described by Ohkawa et al. and Sedlak & Lindsay [32,33], respectively.

### 2.6. Statistical Analysis

The data of the present study was represented as mean ± SEM. The data analysis was performed by one-way analysis of variance (ANOVA) followed by Tukey-Kramer for multiple comparison. The data analyses were carried out by using statistical software, Graphpad Prism 3.0 (San Diego, CA, USA). *p* values < 0.05 were kept as statistically significant.

## 3. Results

### 3.1. Behavioral Assessment

#### 3.1.1. Effect of Thymoquinone on Rotarod Test of Mice

**Figure 1:**
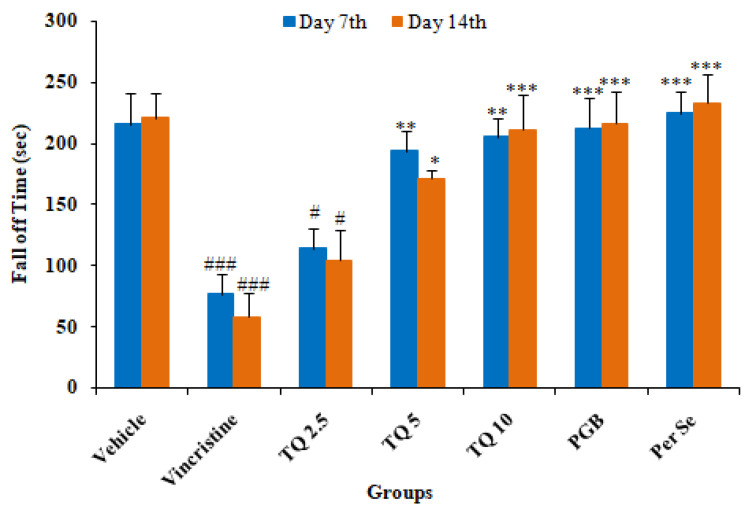
Effects of thymoquinone (TQ) treatment on a rotarod test of mice. All the data are represented as mean ± SEM. Vincristine (VCR) was injected by intraperitoneal (i.p.) route, while thymoquinone (TQ) by oral route (p.o.). The duration of treatment lasted for 14 days. VCR was administered for 10 consecutive days. n, number of animals; Vehicle, 2.5% DMSO; TQ, Thymoquinone; PGB, Pregabalin. # *p* < 0.05, and ### *p* < 0.001 vs. Group I (vehicle). * *p* < 0.05, ** *p* < 0.01, *** *p* < 0.001, vs. Group II (Pathogenic control), considered significant by ANOVA followed by Tukey-Kramer multiple comparison test.

Vincristine administration resulted in a significant (*p* < 0.001) reduction in fall off time on day 7 and day 14 of drug treatment as compared to the control (Figure 1). TQ preventive treatment produced a significant reversal of VCR-induced reduction in fall off time in neuropathic mice. The reversal with a lower dose of TQ (2.5 mg/kg) was statistically significant as compared to the vincristine treated group, however, a higher dose (10 mg/kg) produced a significant (*p* < 0.001) abolition of VCR-induced reduction in fall off time at day 7 and day 14. In the pregabalin (10 mg/kg) treatment group there was significant elevation in fall off time as compared to the pathogenic control (group II). However, TQ per se (10 mg/kg) treatment did not produce significant changes in fall off time on day 7 and 14 as compared to the control group.

#### 3.1.2. Effect of Thymoquinone on Hot Plate Induced Algesia in Mice

**Figure 2:**
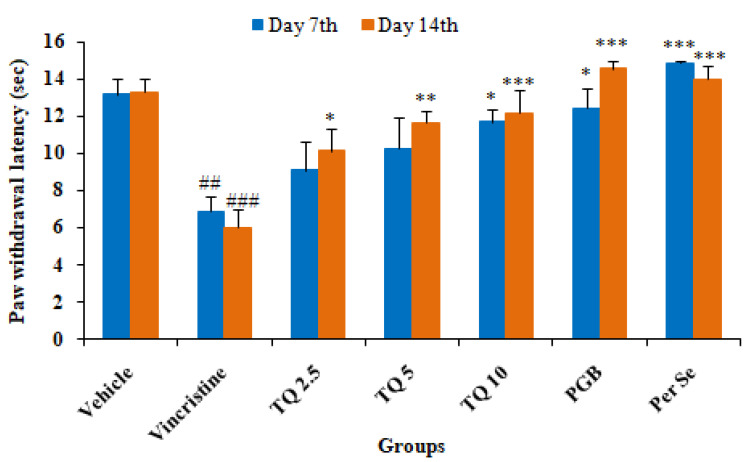
Effects of thymoquinone (2.5, 5, and 10 mg/kg) on thermal hyperalgesia induced by a hot plate in vincristine-induced neuropathy in mice. All the data are represented as mean ± SEM. Vincristine (VCR) was injected by intraperitoneal (i.p.) route, while thymoquinone (TQ) by oral route (p.o.). The duration of treatment lasted for 14 days. VCR was administered for 10 consecutive days. n, number of animals; Vehicle, 2.5% DMSO; TQ, Thymoquinone; PGB, Pregabalin. ## *p* < 0.01, and ### *p* < 0.001 vs. Group I (vehicle). * *p* < 0.05, ** *p* < 0.01, *** *p* < 0.001, vs. Group II (Pathogenic control), considered significant by ANOVA followed by Tukey-Kramer multiple comparison test.

As shown in Figure 2, there was a significant (*p* < 0.01) reduction in paw withdrawal latency in mice following vincristine administration on day 7 and 14 as compared to the control. Concurrent treatment with TQ (2.5, 5, and 10 mg/kg, p.o.) produced a significant reversal of vincristine induced reduction in paw withdrawal latency compared to the pathogenic control. Treatment with TQ 2.5 and 5 mg/kg (group III & IV, respectively) only produced a significant (*p* < 0.05 & *p* < 0.01 respectively) increase in paw withdrawal latency on day 14, but not on day 7 of the treatment. The higher dose of TQ (10 mg/kg) produced a significant reversal in paw withdrawal latency on day 7 and 14 of the hotplate test as compared to the pathogenic control (group II). In group VI (pregabalin, 10 mg/kg), there was a significant increase in paw withdrawal latency on day 7 and 14 as compared to the pathogenic control (group II). TQ per se (10 mg/kg) treatment also increased the paw withdrawal latency on day 7 and 14 of the hotplate test in mice as compared to the pathogenic group.

#### 3.1.3. Effect of Thymoquinone on Cold Plate Induced Algesia in Mice

**Figure 3:**
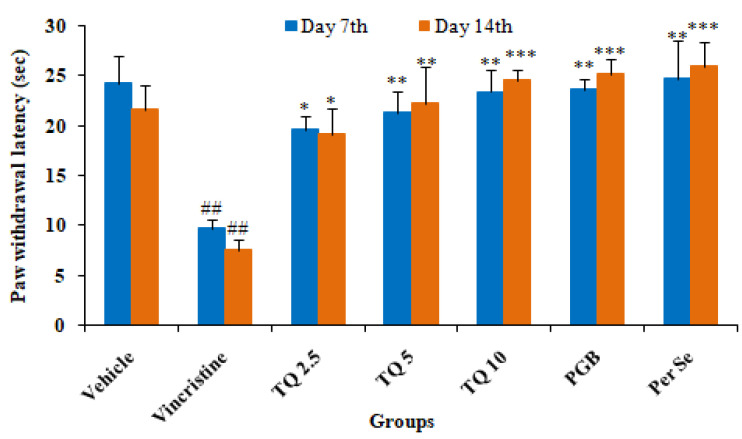
Effects of thymoquinone (2.5, 5, and 10 mg/kg) on thermal allodynia induced by a cold plate in vincristine induced neuropathy in mice. All the data are represented as mean ± SEM. Vincristine (VCR) was injected by intraperitoneal (i.p.) route, while thymoquinone (TQ) by oral route (p.o.). The duration of treatment lasted for 14 days. VCR was administered for 10 consecutive days. n, number of animals; Vehicle, 2.5% DMSO; TQ, Thymoquinone; PGB, Pregabalin. ## *p* < 0.01 vs. Group I (vehicle). * *p* < 0.05, ** *p* < 0.01, *** *p* < 0.001 vs. Group II (Pathogenic control), considered significant by ANOVA followed by Tukey-Kramer multiple comparison test.

The administration of vincristine produced allodynia evidenced by a significant (*p* < 0.01) decrease in the paw withdrawal latency on day 7 and 14 of the cold plate test in comparison to the control. However, TQ (2.5, 5, and 10 mg/kg, p.o.) preventive treatment produced dose-dependent reversal in paw withdrawal latency on day 7 and 14 of the cold plate test in neuropathic mice (group II). Pregabalin (10 mg/kg) also produced significantly (*p* < 0.001) prevented a decrease in paw withdrawal latency in vincristine induced neuropathic mice on the cold plate test. TQ (10 mg/kg, p.o.) per se treatment did not produce significant changes in paw withdrawal latency as compared to the control (vehicle) (Figure 3).

#### 3.1.4. Effect of Thymoquinone on Formalin Test in Mice 

Vincristine induced neuropathic mice exhibited a significant increase in the number of flinches and paw licking duration in response to pain following injection of formalin as compared to the control (vehicle) in both the acute and delayed phase. In the acute phase, the number of flinches and duration of paw licking behavior was more as compared to the delayed phase. The administration of TQ (2.5, 5, and 10 mg/kg, p.o.) produced a significant (*p* < 0.05) diminution in the number of flinches and duration of paw licking in a dose dependent manner in comparison to the pathogenic control (group II). The higher dose (TQ, 10 mg/kg) produced a highly significant (*p* < 0.001) reversal in the number of flinches and duration of paw licking behavior in both the acute and delayed phase of the formalin test. Pregabalin administration produced a significant reduction in the number of flinches and paw licking duration in both the acute and delayed phase of the formalin test as compared to the pathogenic control (group II). However, TQ per se (10 mg/kg, p.o.) treatment also produced a significant reduction in the number of flinches and duration of paw licking in comparison to group II (Table 1).

### 3.2. Biochemical Assessments

#### 3.2.1. Effect of Thymoquinone on MDA Level in Sciatic Nerve Tissue

**Figure 4:**
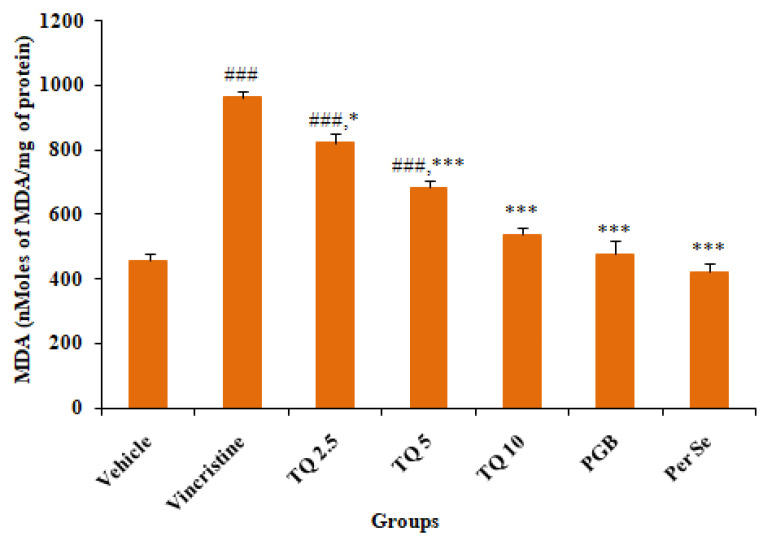
Effects of thymoquinone (2.5, 5 and 10 mg/kg) treatment on MDA levels in sciatic nerve tissues of mice. All the data are represented as mean ± SEM. Vincristine (VCR) was injected by intraperitoneal (i.p.) route, while thymoquinone (TQ) by oral route (p.o.). The duration of treatment lasted for 14 days. VCR was administered for 10 consecutive days. n, number of animals; Vehicle, 2.5% DMSO; TQ, Thymoquinone; PGB, Pregabalin. ### *p* < 0.001 vs. Group I (vehicle). * *p* < 0.05, *** *p* < 0.001, vs. Group II (Pathogenic control), considered significant by ANOVA followed by Tukey-Kramer multiple comparison test.

In comparison to normal control (vehicle), there was a significant (*p* < 0.001) increase in tissue malondialdehyde (MDA) levels, a product of lipid peroxidation in the sciatic nerve of vincristine-induced neuropathic mice (Figure 4). Preventive treatment with TQ (2.5, 5, and 10 mg/kg, p.o.) resulted in dose dependent amelioration of the raised sciatic nerve tissue MDA level, though the reversal with the highest dose of TQ (10 mg/kg) was statistically highly significant (*p* < 0.001) when compared to the pathogenic control (group II). Pregabalin (10 mg/kg, p.o.) treated mice did not show a significant increase in MDA levels of sciatic nerve tissue of vincristine treated mice. No significant alteration in tissue MDA was observed in TQ (10 mg/kg, p.o.) per se treated mice in comparison with the control (vehicle).

#### 3.2.2. Effect of Reduced Glutathione (GSH) Level in Sciatic Nerve Tissue

**Figure 5:**
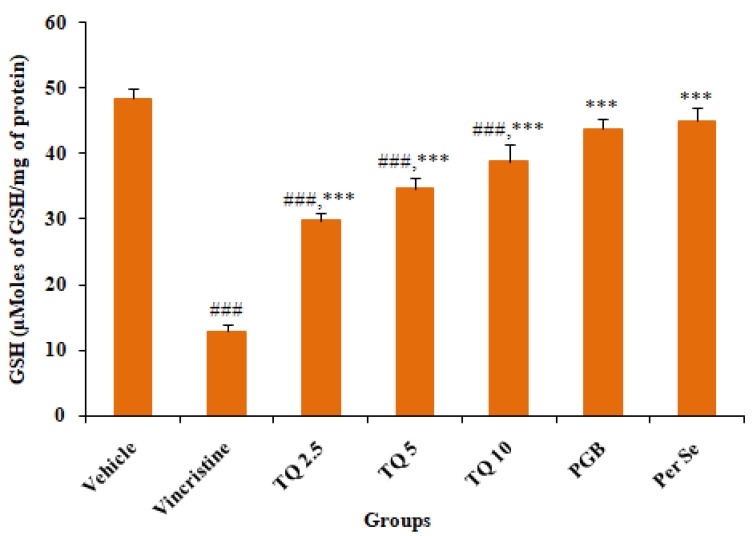
Effects of thymoquinone (2.5, 5 and 10 mg/kg) treatment on GSH levels in the sciatic nerve tissues of mice. All the data are represented as mean ± SEM. Vincristine (VCR) was injected by intraperitoneal (i.p.) route, while thymoquinone (TQ) by oral route (p.o.). The duration of treatment lasted for 14 days. VCR was administered for 10 consecutive days. n, number of animals; Vehicle, 2.5% DMSO; TQ, Thymoquinone; PGB, Pregabalin. ### *p* < 0.001 vs. Group I (vehicle). *** *p* < 0.001, vs. Group II (Pathogenic control), considered significant by ANOVA followed by Tukey-Kramer multiple comparison test.

Figure 5 summarizes the effects of TQ (2.5, 5, and 10 mg/kg, p.o.) treatment on tissue reduced glutathione (GSH) levels in the sciatic nerve tissue of vincristine-induced neuropathic mice. There was a significant (*p* < 0.001) depletion of tissue reduced GSH level in vincristine treated mice as compared to the control (vehicle). Preventive treatment with TQ produced a dose dependent reversal of reduced GSH levels, though the reversal by the higher dose (10 mg/kg) of TQ was statistically more significant in comparison with the pathogenic control (group II). However, pregabalin (10 mg/kg, p.o.) also significantly (*p* < 0.001) restored the depleted GSH levels in vincristine-induced neuropathic mice. TQ per se (10 mg/kg, p.o.) did not produce a significant change in tissue GSH level as compared to the control (vehicle).

#### 3.2.3. Effect of Thymoquinone on IL-6 Level in Serum

**Figure 6:**
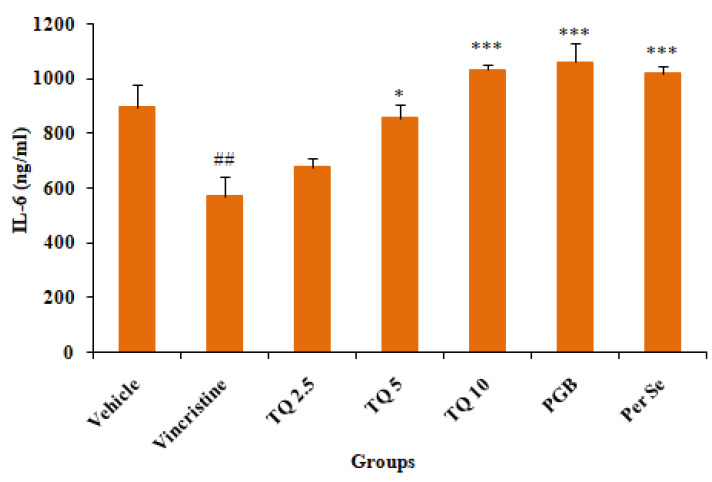
Effects of thymoquinone (2.5, 5 and 10 mg/kg) treatment on serum IL-6 levels in mice. All the data are represented as mean ± SEM. Vincristine (VCR) was injected by intraperitoneal (i.p.) route, while thymoquinone (TQ) by oral route (p.o.). The duration of treatment lasted for 14 days. VCR was administered for 10 consecutive days. n, number of animals; Vehicle, 2.5% DMSO; TQ, Thymoquinone; PGB, Pregabalin. ## *p* < 0.01vs. Group I (vehicle). * *p* < 0.05, *** *p* < 0.001, vs. Group II (Pathogenic control), considered significant by ANOVA followed by Tukey-Kramer multiple comparison test.

Vincristine treatment showed a significant (*p* < 0.001) reduction in serum IL-6 levels as compared to the control (vehicle). TQ (2.5, 5, and 10 mg/kg, p.o.) preventive treatment produced a dose-dependent reversal of serum IL-6 depletion in vincristine treated mice, though the reversal by the lower dose (2.5 mg/kg, p.o.) of TQ was statistically insignificant. Concurrent treatment with pregabalin (10 mg/kg, p.o.) also produced significant elevation in serum IL-6 levels in vincristine-induced neuropathic mice as compared to the pathogenic control (group II). Besides, TQ per se (10 mg/kg, p.o.) also significantly increased serum IL-6 levels as compared to the control (vehicle) (Figure 6).

## 4. Discussion

Peripheral neuropathy continues to be one of the most common and debilitating side effects of vincristine in cancer patients. Currently, limited pharmacological agents are available for the prevention and management of neuropathic pain induced by chemotherapeutic agents, hence dose reduction or the discontinuation of therapy with drugs such as vincristine and platinum compounds are advocated in a number of patients [34]. The binding of vincristine with β tubulin in neurons and the impairment of microtubule polymerization are attributed to the neurotoxic and anticancer effects. Furthermore, vincristine also results in the degeneration of axons by producing highly reactive free radicals and altering the release of inflammatory cytokines [35]. 

In the present study, the neuroprotective effect of TQ was evaluated against VCR-induced neuropathy in mice. As reported previously, mice exhibited neuropathy following a VCR injection, evident from a significant reduction in performance on the rotarod test and pain threshold in models of hyperalgesia, and the formalin test [16]. VCR-treated mice showed a loss of grip strength on the rotarod test, reduced pain threshold, and increased oxidative stress in the sciatic nerve in this study. Pregabalin, used as a standard drug, showed significant protection against VCR-induced neuropathic pain in mice, possibly due to inhibitory action on the release of glutamate and sensory neuropeptides in the synapse by binding to the alpha2-delta (α2–δ) subunit of voltage-gated calcium channel [36]. Treatment with TQ (2.5, 5, and 10 mg/kg) produced a significant increase in grip strength on the rotarod and threshold of nociception in VCR treated mice on the hot plate, cold plate, and the formalin test, as well as a significant reversal of oxidative stress in sciatic nerve tissues and serum IL-6 levels.

Vincristine causes paresthesia, sensory impairment, and, to a lesser extent, motor deficits in a dose and duration-dependent manner [37]. The rotarod performance test is a widely used experimental paradigm for assessing progressive loss of sensory and motor functions in rodents [38]. Neuropathic mice exhibited impairment of sensory-motor function, manifested as a significant decline in performance on the rotarod test, as reported previously [39]. There was a severe decline in motor performance on the rotarod in adult rats following a once-daily intravenous VCR (200 µg/kg) injection for two weeks [40]. Contrary to this, it has been reported that rotarod performance statistically differed insignificantly either between wild type and SARM1 (sterile alpha and TIR motif containing protein 1) knockout mice, or at the baseline and after VCR treatment, indicating the absence of gait disturbance [3]. TQ therapy restored the VCR-induced loss in rotarod performance in mice, which is consistent with previous findings of a considerable improvement in sensory and motor function in dorsal root ganglia (DRG) cell cultures treated with cisplatin [41]. TQ therapy may improve motor performance due to its neuroprotective and antioxidant effects, as it has been found to reverse rotenone-induced motor impairment in rats [42].

Pain behavior in cancer patients due to chemotherapeutic agent progress as acute pain syndrome, which may often necessitate dose reduction or the cessation of therapy that negatively affects the outcome of therapy and the re-emergence of cancer. The behavioral alterations such as sensory-discrimination, affective motivation, and cognitive function are used for the assessment of pain in rodents. Thermal algesia such as hot and cold plates is most commonly used for the screening of analgesics and is considered as a gold standard model for pain testing in rodents. Chemotherapy-induced neuropathy results in a delayed response to cold and heat stimuli in rodents. Concurrent TQ therapy reversed the VCR-induced increase in pain-associated behavior in mice on both hot and cold plates in a dose-dependent manner. The findings of the present study are in agreement with previous studies that reported amelioration of neuropathic pain induced by mechanical injury to the spinal cord in rats [25]. Furthermore, TQ’s neuroprotective and antinociceptive benefits in the chronic model of neuropathy in animals were also linked to its reduction of oxidative stress and inhibition of microglia activity [43]. Consistent with our findings, another study found that Pregabalin treatment reduced neuropathic pain in rats after chronic constriction injury [44].

Formalin-induced nociception has been widely used in animal studies [45], which produces acute inflammatory pain often associated with tissue injury. Administration of 2.5% formalin induces concentration dependent hypersensitivity through multiple mechanisms, showing resemblance to spinal nerve injury associated neuropathic pain in animals and clinical pain as compared to other models [46]. The pain associated with formalin is typically biphasic, with two peaks: an acute phase (0 to 5 min) and a persistent delayed phase (15 to 20 min) induced by localized inflammation of tissue following the formalin injection [47]. In addition, the acute phase of formalin-induced nociception is characterized by peripheral activation of C-fibres, whereas the delayed phase is a result of tissue injury. Centrally acting analgesics reduce both the acute and delayed phases of pain response, whereas peripherally acting drugs inhibit only the delayed phase [48]. Our results showed that the injection of formalin induces pain-associated responses, such as an increase in the number of flinches and paw licking duration in both the acute (0–5 min) and delayed phase (15–20 min). TQ exhibited a reduction in nocifensive behaviors in both phases of formalin-induced algesia in mice, though it was more significant in the acute phase. The peripheral analgesic action of TQ is in agreement with a previous study reporting an antinociceptive effect in neuropathic pain induced by spinal cord injury in rats [25]. In addition, the antinociceptive effects of TQ in the hotplate, cold plate, and delayed phase of the formalin test indicate plausible central analgesic actions, which is in line with previous studies reporting neuronal protection in cerebral ischemia induced by reperfusion injury in rats [49].

There is substantiating evidence suggesting the involvement of impaired balance between the generation of reactive oxygen species (ROS) and the detoxifying ability against the noxious effects of ROS, leading to cellular oxidative stress, a predictable key factor in the pathogenesis of peripheral neuropathy [35]. Furthermore, it has been reported that neurons are more vulnerable to oxidative damage due to their limited antioxidant capacity and high oxygen demand [50]. In the current study, VCR injection significantly increased cellular oxidative stress, as demonstrated by an increase in MDA, due to accelerated membrane lipid peroxidation and a reduction in reduced glutathione (GSH) in sciatic nerve tissue. Glutathione is the most abundant intracellular thiol antioxidant with a low molecular weight, and it resides predominantly in a reduced form (90–95%) in healthy cells. It produces a low-oxygen environment for the functioning of various enzymes in cells and is essential for metabolic defensive functions, such as decreasing hydroperoxide, detoxifying xenobiotics, and scavenging of free radicals [51]. Thus, measuring reduced GSH could be a useful indicator of cellular oxidative stress caused by exogenous compounds. Oxidative stress has been recognized as a mainstay of diabetes-induced complications such as vascular and neurologic dysfunction, leading to impaired functioning of motor and sensory neurons due to intraneural hypoxia [52]. Mitochondrial oxidative stress has been associated with a diverse range of disorders, including neurologic pain. Interestingly, it has been reported that there was an increase in mitochondrial viability and a reduction in apoptosis following treatment with TQ, suggesting the antioxidant and antiapoptotic action against kidney injury in rats [53]. Concurrent TQ therapy reversed the VCR-induced increase in MDA and depleted reduced GSH in mouse sciatic neurons, which is commensurate with a previous study that demonstrated the amelioration of liver damage in diabetic rats by the control of oxidative stress and cellular inflammation [54].

Cellular inflammation is the hallmark of nerve damage, and the administration of VCR results in an increased release of cytokines. Interleukin-6 (IL-6) is a multifunctional cytokine with anti-inflammatory and pro-inflammatory actions through the trans and classic signaling pathways, respectively [9]. Until now, according to our knowledge, the majority of researchers have mostly concentrated on the classic signaling pathway-mediated effects of IL-6 in neuropathy. In contrast, the majority of the reported literature focused on IL-6’s pro-inflammatory action in mediating neuropathy, while the numerous beneficial effects of IL-6, such as increased blood flow, regulation of lipid metabolism, relief of long-term inflammation, and regeneration of nerve fibers, received less attention [55]. Our results showed that VCR (0.1 mg/kg, i.p.) administration produced a significant decrease in serum IL-6 levels on day 14 following 10 days of consecutive administration in mice. There is evidence of an increase in the release of IL-6 both in the proximal and distal regions following an injury to the sciatic nerve [56]. Nevertheless, following 12 to 24 h after nerve damage, the distal level of IL-6 declines rapidly [57]. TQ treatment resulted in a dose-dependent increase in serum IL-6 levels in VCR-treated mice. Therefore, it is plausible that the administration of VCR might diminish the neuroprotective impact of IL-6 through classic signaling-mediated anti-inflammatory action, which was reversed by TQ administration. Intriguingly, it has been discovered that the expression of the IL-6 receptor (which facilitates classic signaling) increases with age in intact nerves [58]. According to the findings of our study, therapy with VCR causes a reduction in the anti-inflammatory action of IL-6 that occurs in response to nerve injury. This reduction was prevented by concurrent treatment with TQ, which led to a reversal of the VCR-induced fall in serum IL-6 levels. Supporting this notation, a study found that the administration of low-dose pulsatile IL-6 could exert neuroprotection against diabetes-induced peripheral neuropathy [55]. In this study, there was an increase in IL-6 levels following 14 days of treatment with PGB. This could be attributed to the fact that IL-6 is a pleiotropic molecule and is regarded both as pro- and anti-inflammatory in nature. The increase in IL-6 levels could have contributed to the antioxidant effect of PGB which is also supported by the reversal of VCR mediated alteration in MDA and GSH levels. Our results are consistent with earlier research showing that IL-6 suppresses mitochondrial ROS through increasing the activity of nuclear factor erythroid 2-related factor 2 (Nrf2) [59].

Preventive treatment with TQ (2.5, 5, and 10 mg/kg, p.o.) for 14 days produced a dose-dependent reversal of VCR-induced pain behavior in mice, with the higher doses (5 and 10 mg/kg, p.o.) producing a very significant impact on biochemical and behavioral parameters. Although not statistically significant, the lower dose of TQ (2.5 mg/kg, p.o.) also reversed VCR-induced changes in assessed parameters. This may be due to the shorter duration of drug treatment, which should be further investigated in a long-term study of treatment for VCR-induced neuropathic pain. The study shows that oxidative stress is involved in VCR-induced neuropathic pain behavior in mice and its modulation by TQ preventive treatment. There are limited studies investing the effect of TQ on VCR-induced peripheral neuropathy and to the best of our knowledge this is the first study investigated the preventive effect of TQ using this model. However, the study only includes a small number of oxidative stress measures in tissue, suggesting that additional research is required to evaluate the preventative effect of TQ in other neuropathic pain models. 

## 5. Conclusions

The present study takes precedence in establishing TQ as a potential add-on therapy for various forms of neuropathic pain, particularly those arising from the administration of chemotherapeutic agents such as VCR. TQ’s antinociceptive effects are evidenced by a significant increase in grip strength on the rotarod, as well as reduced pain sensitivity on the hotplate, cold plate, and formalin tests; a reduction in free radicals mediated sciatic nerve damage, and a reversal of serum IL-6 levels. Notably, the research has shown that IL-6 is not only pro-inflammatory but also has anti-inflammatory properties via classic signaling pathways and causes improved blood flow and control of lipid metabolism. The findings of this research prove to be extremely important in demonstrating another, more positive aspect of these processes. Because pregabalin is widely used as a standard treatment for neuropathic pain, researchers may investigate whether combining it with TQ is beneficial in the near future. In addition to improving blood flow, controlling lipid metabolism, lowering chronic inflammation, and stimulating brain regeneration, IL-6 plays a role in all of these processes.

## Figures and Tables

**Table 1:** Effects of thymoquinone (2.5, 5 and 10 mg/kg) treatment on the acute and delayed phase of nociception induced by formalin in neuropathic mice.

Treatment (n = 5)	Dose	Acute Phase		Delayed Phase	
		No. of Flinches	Licking Duration (Sec)	No. of Flinches	Licking Duration (Sec)
Vehicle	10 mL/kg, p.o.	4.4 ± 0.812	44.2 ± 8.49	1.4 ± 0.509	11.82 ± 3.251
Vincristine	0.1 mg/kg, sc	161.0 ± 4.701 ###	254.29 ± 27.55 ###	38.2 ± 6.98 ###	34.47 ± 2.741 ###
TQ 2.5	2.5 mg/kg, p.o.	134.8 ± 9.521 ###	226.76 ± 18.81 ###	21.4 ± 5.202 #,*	23.96 ± 1.172 ##,*
TQ 5	5 mg/kg, p.o.	106.2 ± 4.454 ###,**	172.9 ± 18.50 ###,*	19.0 ± 2.51 #,*	20.21 ± 0.785 ***
TQ 10	10 mg/kg, p.o.	80.8 ± 5.544 ###,***	147.43 ± 8.03 ##,**	12.4 ± 2.315 ***	14.32 ± 1.261 ***
PGB	10 mg/kg, p.o.	78.0 ± 17.595 ###,***	142.77 ± 18.08 ##,**	8.4 ± 1.887 ***	12.14 ± 1.599 ***
Per se	10 mg/kg, p.o.	80.0 ± 5.568 ###,***	132.2 ± 16.18 #,***	5.6 ± 1.965 ***	16.14 ± 2.018 ***

All the data are represented as mean ± SEM. Vincristine (VCR) was injected by intraperitoneal (i.p.) route, while thymoquinone (TQ) by oral route (p.o.). The duration of treatment lasted for 14 days. VCR was administered for 10 consecutive days. n, number of animals; Vehicle, 2.5% DMSO; TQ, Thymoquinone; PGB, Pregabalin. # *p* < 0.05, ## *p* < 0.01, ### *p* < 0.001 vs. Group I (vehicle). * *p* < 0.05, ** *p* < 0.01, *** *p* < 0.001, vs. Group II (Pathogenic control), considered significant by ANOVA followed by Tukey-Kramer multiple comparison test.

## Data Availability

The raw data supporting the conclusions of this article will be made available to any qualified researcher on request.
